# Ovarian Adenomatoid Tumor Coexisting with Mature Cystic Teratoma: A Rare Case Report

**DOI:** 10.1155/2017/3702682

**Published:** 2017-09-11

**Authors:** Mingxia Shi, Firas Al-Delfi, Majd Al Shaarani, Kurt Knowles, James Cotelingam

**Affiliations:** ^1^Department of Pathology and Translational Pathobiology, Louisiana State University Health Science Center-Shreveport, 1501 King's Highway, Shreveport, LA 71130, USA; ^2^Pathology and Laboratory Medicine, Dr. Sulaiman Al Habib Medical Group, P.O. Box 505005, Dubai Healthcare City, Dubai, UAE

## Abstract

Adenomatoid tumor of the ovary is rare, and so are collision tumors in this location. The most common histological combination of ovarian collision tumors is the coexistence of mature cystic teratoma with ovarian cystadenoma or cystadenocarcinoma. Presented herein is a rare case of ovarian adenomatoid tumor found incidentally and coexisting with mature cystic teratoma. A 44-year-old woman presented with a one-year history of intermittent right-sided pelvic pain. Ultrasound evaluation revealed a heterogeneous cystic mass in the right ovary, and a clinical diagnosis of teratoma was made. The patient subsequently underwent a right salpingo-oophorectomy. Pathological examination revealed a mature cystic teratoma and coexistent adenomatoid tumor. The two tumors were separate and no transitional features were recognized histologically. To our knowledge, no previous report of coexistence of these two tumors has been reported. Both tumors are benign and completely excised; therefore no adverse consequences are expected.

## 1. Introduction

Adenomatoid tumors (ATs) are benign lesions of mesothelial origin. Commonly arising in the male genital tract, they are responsible for 30% of all paratesticular masses [1] and are the most common tumor of the epididymis and spermatic cord. In women, they are commonly found in the myometrium and are the most common benign tumor of the fallopian tubes [2]. AT is uncommon in the ovary, and only a very limited number of cases have been recorded [3–6].

Mature cystic teratoma (MCT), also known as dermoid cyst, is a germ cell neoplasm composed of well-differentiated derivations from at least two of the three germ cell layers and accounts for approximately 15% of ovarian neoplasms. MCT tend to be identified in young and middle-aged females [7]. These tumors are usually benign and unilateral but can be bilateral in 10–15% of cases [8].

A collision tumor is the coexistence of two adjacent, abutting, but histologically distinct tumors without histological admixture in the same tissue. Compared to other organs, ovarian collision tumors are rare and most commonly consist of a benign MCT and an ovarian cystadenoma or cystadenocarcinoma. Rarer histologic combinations have been also reported [9, 10].

We report an extremely rare presentation of collision tumor in the ovary consisting of an MCT and AT.

## 2. Case Presentation

A 44-year-old (G0) female presented to the gynecology clinic complaining of intermittent right-sided pelvic pain that radiated to her back for one year. She was postmenopausal from age of 37. There was a past surgical history of left oophorectomy and right ovarian cystectomy for “dermoid cyst.” A subsequent pelvic ultrasound was performed. The right ovary measured approximately 3.6 × 3 × 3.2 cm. In the right adnexal region dense acoustical shadowing was appreciated. The right ovary was nearly completely replaced by a heterogeneous cystic mass with hyperechogenic lines and spots, which are most compatible with ovarian teratoma. The left ovary was absent. No free pelvic fluid was noted. Robotic right salpingo-oophorectomy was performed and entire specimen was sent to pathology for examination.

Grossly, the specimen consisted of right fallopian tube and ovary. Sectioning through the ovary revealed a 2.8 × 1.3 × 1.5 cm cystic lesion containing greasy material, hair, and teeth, characteristics of teratoma. A circumscribed tan-white solid nodule of 0.8 × 0.8 × 0.7 cm was also noted in the adjacent ovarian tissue. The fallopian tube was grossly unremarkable.

**Figure 1:**
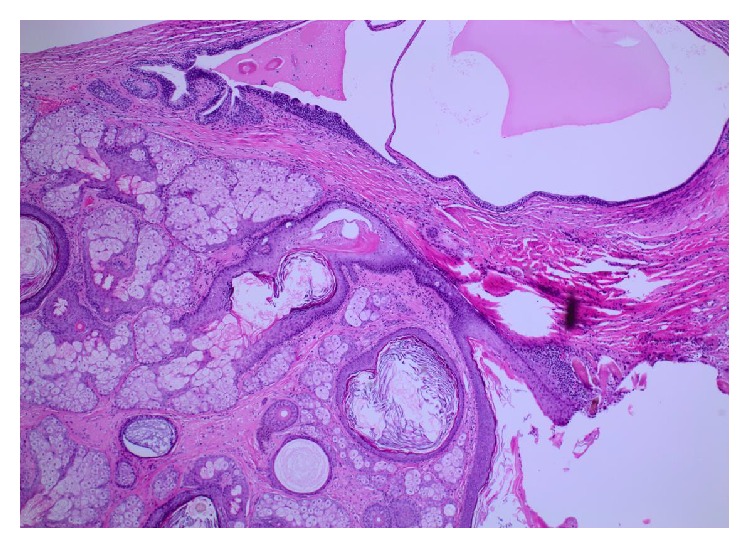
*Mature cystic teratoma* (H&E ×4), showing keratinized squamous epithelium, sebaceous glands, and hair follicles. Respiratory epithelium (left upper) is also present.

**Figure 2:**
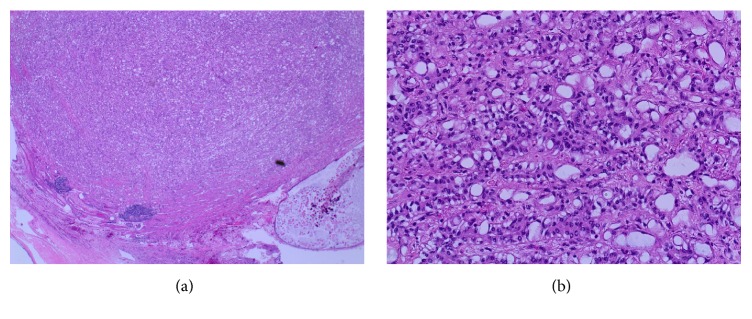
*Adenomatoid tumor.* (a) shows well circumscribed tumor with few peripheral lymphoid aggregates (H&E ×4). (b) shows major pattern of adenoid and glandular cells with markedly vacuolated cytoplasm (H&E ×20).

**Figure 3:**
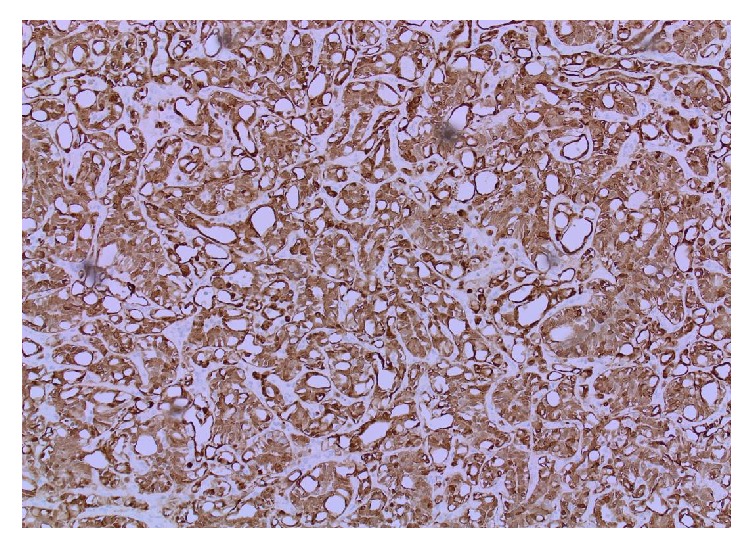
*Positive immunoreactivity to calretinin* (H&E ×20). Adenomatoid tumor with cells displaying strong and diffuse positivity for calretinin-index of mesothelial origin.

Microscopically, the ovarian cyst was lined by stratified squamous epithelium associated with keratinous material, hair follicles, and sebaceous glands (Figure 1). Respiratory epithelium, cartilage, and fatty tissue were also present. No evidence of immature elements was observed. The solid nodule showed an unencapsulated but circumscribed tumor with several lymphoid aggregates noted towards the periphery of the tumor (Figure 2(a)). The tumor was composed of clefts and spaces lined by cuboidal, low columnar, and flattened epithelial-like cells, predominantly arranged in adenoid and glandular patterns. Some tumor cells exhibited marked vacuolation (Figure 2(b)). Cytologic atypia and mitoses were absent. On serial sections, this solid tumor was situated 0.2 cm from the closest teratomatous components containing hair follicles and respiratory epithelium. The circumscribed solid tumor was strongly positive for pancytokeratin (AE1/AE3), calretinin (Figure 3), and D2-40, weakly positive for WT1, and negative for CD31. A final diagnosis of AT coexisting with MCT of the ovary was rendered.

## 3. Discussion

Most reports of ovarian adenomatoid tumor are in patients in the third and fourth decades. The lesions usually are found in the hilus of the ovary as incidental findings. ATs are rarely larger and symptomatic and may extend into and replace the ovarian parenchyma [3]. They have benign behavior, and there are no reports of recurrence or malignant transformation. Surgical excision is curative.

Morphologic patterns of AT include adenoid, angiomatoid, microcystic, glandular, trabecular, tubular, and plexiform. The most frequent types are adenoid and angiomatoid [2, 3, 11]. With the exception of tubal and, possibly, ovarian sites, most genital tract ATs exhibit an infiltrative pattern. AT with a diffuse, infiltrative growth pattern may cause a diagnostic dilemma. Hence, a differential diagnosis of lymphangioma, hemangioma, epithelioid hemangioendothelioma, signet-ring cell adenocarcinoma, and yolk sac tumor should be considered and can be sorted out on Immunohistochemical examination. It is important to be aware of this benign subtype of mesothelial tumor and recognize these tumors in the frozen section as this can avoid more aggressive surgery. Our case showed a well-demarcated tumor with major adenoid and glandular patterns and minor angiomatoid and microcystic patterns. Lymphoid aggregates are useful diagnostic features of male genital tract ATs but are infrequent (only 13%) in female genital tract sites [12]. Our case showed several lymphoid aggregates in the periphery of the ovarian AT. Immunohistochemistry in our case revealed strong positivity of pancytokeratin (AE1/AE3) and mesothelial markers (calretinin and D2-40), confirming the diagnosis of adenomatoid tumor. The WT-1 antibody showed weaker staining intensity and also demonstrated background staining. The limited diagnostic utility of WT-1 in AT of female genital tract sites due to strong background staining was also demonstrated by Sangoi et al. [12]. Their study also showed cytoplasmic immunoreactivity for CK5/6, a well-known marker for mesothelial associated neoplasm, in only 16% of female and 8% of male with ATs. Absent expression of endothelial marker of CD31 in our case argues against the diagnosis of a lymph-vascular neoplasm.

The ectodermal tissue in this right ovarian MCT was composed of squamous epithelium with cutaneous appendages, and the mesodermal component was represented by cartilage and fat. The only endodermal tissue present was respiratory epithelium. Given the previous surgical history of left oophorectomy for dermoid cyst (performed at another institution), our case represents an example of bilateral MCT.

We report an extremely rare coexistence of a collision AT and MCT in the right ovary. In our case, the AT and MCT were separated by intervening fibrous tissue. No transitional features were observed on histological examination. Although it is plausible that germ cells from the MCT could differentiate into the mesothelial epithelium that give rise to adenomatoid tumor, our findings suggest that these two tumors coexisted independently.

## 4. Conclusion

To the best of our knowledge this is the first reported case of coexistent AT and MCT in the ovary. Both these tumors were benign and therefore no adverse consequences are expected for the patient. The potential existence of ovarian collision tumors is further reason for careful gross and microscopic examination of tissue at this site.
